# Reply to Hornibrook, J. Comment on “Tabet et al. Vestibular Migraine versus Méniere’s Disease: Diagnostic Utility of Electrocochleography. *Audiol. Res.* 2023, *13*, 12–22”

**DOI:** 10.3390/audiolres14030042

**Published:** 2024-05-30

**Authors:** Issam Saliba, Paul Tabet

**Affiliations:** 1 Division of Otorhinolaryngology and Head & Neck Surgery, University of Montreal, Montreal, QC H3C 3J7, Canada; 2 University of Montreal Health Center (CHUM), Department of Otorhinolaryngology and Head & Neck Surgery, Montreal, QC H2X 3E4, Canada; 3 University of Montreal Health Center Research Center (CRCHUM), Montreal, QC H2X 0A9, Canada

We appreciate the comments made by Hornibrook (2024) [1] on our paper reporting on the Vestibular Migraine (VM) versus Ménière’s Disease (MD): Diagnostic Utility of Electrocochleography (ECochG) [2].

We agree that the ECochG to make a distinction, is becoming unfashionable with the increased use of magnetic resonance imaging (MRI) inner-ear imaging. However, the access to 3 Tesla MRI for hydrops identification is not as easy as in our university hospital center. Majority of hospital doesn’t have this privilege yet, whereas ECochG is easily accessible and affordable. Therefore, we believe that ECochG still plays an important role in the management of Ménière’s disease.

In his comment, Hornibrook reported that the ECochG technique employed was with a remote (eardrum) electrode and a click stimulus and that the diagnostic sensitivity from clicks is vastly inferior to tone bursts. In general, to collect responses in ECochG, 2 recording techniques transtympanic (TT) and extratympanic (ET) are mainly applied. The TT procedure requires insertion a needle electrode through the tympanic membrane to get a direct contact between the electrode tip and the cochlear promontory. In the ET methods, the electrode is directly in contact with the tympanic membrane, collecting responses without any physiological barrier making this technique very attractive clinically as it is less invasive.

What are the indications for the one or the other techniques? When hearing loss is not exceeding 60 dB on any frequency of 500 Hz or more, an ET is used. On the other hand, if there is hearing loss for more than 60 dB on any of these frequencies, a TT ECochG is applied. Although the TT ECochG collects amplitude responses approximately 4 times greater than ET ECochG measurements, patients reported a tolerable pain and could be exposed to potential minor complications such as residual tympanic perforation or bleeding [3].

Hornibrook et al. compared the summating potential (SP) amplitude value by a TT-TBS (tone burst) on different frequencies from 500 Hz to 8 KHz, to intratympanic gadolinium MRI of the inner ear showing the endolymphatic hydrops and to ET SP/AP amplitude ratio (AP: action potential); they concluded that SP amplitude at 1 KHz is the most sensitive test among ECochG tests [4]. In view of this very interesting information, we added to our protocol the measurement of the SP amplitude value by a TT-TBS. Hornibrook unaware of this information, assumed in his comment that audiologists have not employed the tone burst stimulus ECochG because it requires specialty co-operation and customized equipment that cannot be achieved by the standard commercial systems used by audiologists. However we realized throughout the period where we included the TT-TBS in our protocol that these data of TT-TBS were not as helpful as Hornibrook reported; that’s why we didn’t included their analysis in our published article to report utility of ECochG in VM and MD [2].

Faced with this divergence, and in order to eliminate any form of confusion for our esteemed readers, we decided to evaluate the sensitivity and the specificity of SP/AP AUC (area under the curve) ratio by a TT-CS (click-stimulus), SP/AP AUC ratio by an ET-CS and SP amplitude value by a TT-TBS in regard of MD diagnosis. This analysis was recently published [5]. We reported results about ninety-five patients that met the inclusion criteria for electrocochleography (ECochG) testing in our tertiary care center. The sensitivity and the specificity of SP/AP area ratio by a TT-CS were 88.5% and 70.0%, respectively [5]. On the other hand, the sensitivity and specificity for the SP amplitude value by a TT-TBS were 60.0% and 55.6%, respectively. SP/AP area ratio by TT-CS was statistically better than SP amplitude value by TT-TBS to detect MD disease (*p* = 0.016). However, no difference was identified between SP/AP area ratio by ET-CS and SP amplitude value by a TT-TBS (*p* = 0.573), as well no difference was found between the SP amplitude value by TT-TBS and the SP/AP amplitude ratio by ET-CS (*p* = 0.592). Consequently, SP amplitude at 1 KHz is not the most sensitive test among ECochG tests.

ECochG would be extremely useful in the diagnosis of MD if we use the SP/AP area ratio (sensitivity: 88.5%) [5]; therefore, it changes the bad reputation of ECochG sensitivity using SP/AP amplitude ratio (sensitivity: 51.7%) for the diagnosis of MD. Of course, larger prospective studies with normal healthy subjects are recommended to generalize these findings.
